# Racial differences in pathologic complete response rate and clinical outcomes following neoadjuvant chemotherapy for breast cancer

**DOI:** 10.1007/s10549-026-07972-y

**Published:** 2026-05-05

**Authors:** Ashley Matusz-Fisher, Chad A. Livasy, Erin E. Donahue, Lejla Hadzikadic-Gusic, Ashley Y. Martin, Michelle L. Wallander, Brittany Neelands, Dhvani Buch, Alicia Griffin, Arielle L. Heeke, Antoinette R. Tan, Richard L. White

**Affiliations:** 1 Department of Solid Tumor Oncology and Investigational Therapeutics, Atrium Health Levine Cancer, Wake Forest University School of Medicine, Charlotte, NC USA; 2 Department of Pathology, Atrium Health Carolinas Medical Center, Charlotte, NC USA; 3 Department of Biostatistics and Data Sciences, Atrium Health Levine Cancer, Charlotte, NC USA; 4 Division of Surgical Oncology, Department of Surgery, Atrium Health Levine Cancer, Wake Forest University School of Medicine, Charlotte, NC USA; 5 Clinical Trials Office, Atrium Health Levine Cancer, Charlotte, NC USA

**Keywords:** Pathologic complete response (pCR), Race, Racial disparities, Triple-negative, Neoadjuvant chemotherapy (NAC), Overall survival (OS)

## Abstract

**Purpose:**

Neoadjuvant chemotherapy (NAC) is commonly used in early-stage breast cancer. A complete pathologic response (pCR) after NAC is associated with improved outcomes. This study investigated differences in pCR and clinical outcomes by race.

**Methods:**

A single-institution, retrospective chart review identified patients with early-stage breast cancer who received NAC between January 1, 2010, and December 31, 2017. Associations between race and pathologic and clinical outcomes were evaluated using multivariable logistic regression and Cox proportional hazard models. Kaplan–Meier estimates and log rank tests assessed differences in recurrence-free survival (RFS) and overall survival (OS).

**Results:**

A total of 532 patients with breast cancer of all receptor subtypes were identified; 323 (60.7%) White, 188 (35.3%) Black and 21 (3.9%) other/unknown. The pCR rate was different between the 3 race categories; White 27.2%, Black 19.1% and other/unknown 9.5% (*P* = 0.03). In multivariate analysis, pCR rates were higher in White versus Black patients (*P* = 0.02). Patients with triple-negative disease demonstrated the largest difference in pCR (White 44.3% versus Black 27.1%; *P* = 0.04). Black patients had inferior OS compared to White patients (*P* = 0.03). There was no difference in RFS by race (*P* = 0.07).

**Conclusion:**

Black patients demonstrated a lower pCR rate compared to White patients, and this was more pronounced in the triple-negative subgroup. There was no difference in RFS by race, but OS was inferior among Black patients. It is possible that the lower pCR rate in Black patients may contribute to lower OS; however, more investigation is needed to explain these differences.

## Introduction

Breast cancer is a heterogeneous disease that affects people across different ethnicities, race, age, geographic location, education level and economic status. It is estimated that 319,750 people will be diagnosed with a new invasive breast cancer in 2025 [1]. There have been major improvements in breast cancer treatments over the past few decades; however, disparities in outcomes among racial and ethnic groups still persist [2–6]. Black women have a 38% higher mortality compared to White women, despite an incidence rate that is 5% lower. This difference in mortality is largest in young patients and narrows as women age [7]. Black women are more likely to have triple-negative breast cancer (TNBC), which negatively impacts overall outcomes and mortality rates, as TNBC portends a poorer prognosis compared to other subtypes [8, 9]. However, it has been reported that Black patients have the lowest survival regardless of subtype [7]. While socioeconomic differences have been shown to play a role in these differences, data also suggest that tumor biology contributes as well [10, 11].

Neoadjuvant chemotherapy (NAC) can offer multiple advantages, and therefore, is commonly administered to many patients with early-stage breast cancer. Some of these advantages include the ability to downsize the tumor in the breast and/or axilla prior to surgery as well as guide adjuvant treatment decisions based on the pathologic response at the time of surgery [12]. Furthermore, the residual cancer burden (RCB) and achievement of a pathologic complete response (pCR) at the time of surgery has been shown to be prognostic in multiple studies, including our previously published institutional data [13–20]. Therefore, we have elected to investigate this further. Identifying factors that are associated with higher (or lower) rates of pCR, such as race, will be helpful in optimizing treatment and oncologic outcomes for all patients with breast cancer.

Based on the most recent studies, patients with TNBC (pCR rate 50–65%)[21] and human epidermal growth factor receptor 2 (HER2)-positive breast cancer (pCR rate 35–55%) [22, 23] have a higher pCR rate compared to hormone receptor (HR)-positive, HER2-negative tumors (10–20%) [24]. In addition to receptor subtype, the pCR rates can be affected by various other factors, one of these being race. Previous studies have shown that the impact of race on pCR and clinical outcomes can vary, even among the different breast cancer receptor subtypes [10, 25–28]. Some of these studies do not show any difference in pCR rates among race, although many of these are older and perhaps less generalizable with the addition of HER2-targeted therapies and reliance on genomic testing. Other studies show a significant difference in pCR rates among race, but the receptor phenotype with the most significant differences can vary. Furthermore, with the addition of immunotherapy into the neoadjuvant treatment landscape with TNBC, there is published data to suggest no significant differences in pCR according to race [29]. It is essential to understand factors that impact pCR differences at the molecular level and across racial and socioeconomic groups. As the treatment landscape evolves and improves, there remains a risk of further disparities. One avenue to keep abreast of these ongoing disparities is by analyzing institutional and real-world data, as it most closely reflects the patient population and actual clinical practice. Our results are a benchmark for future institutional studies that will be planned to evaluate these disparities over time and with newer neoadjuvant regimens.

Herein, this retrospective single-institution study sought to evaluate pCR and clinical outcomes associated with NAC in all breast cancer receptor subtypes by race.

## Methods

### Study population

A retrospective chart review identified a cohort of 532 female patients diagnosed with early-stage breast cancer who received NAC followed by surgery at Atrium Health Levine Cancer between January 1, 2010, and December 31, 2017. All receptor phenotypes were included. Inclusion criteria were age > 18 years, clinical stage I-III, and receipt of ≥ 75% of intended NAC regimen. Men and patients with clinical stage IV disease were excluded. This study was approved by the Institutional Review Board (IRB 00083102).

### Variables

Demographic and clinicopathological characteristics recorded from the electronic medical record system included age, sex, race, ethnicity, menopausal status, genetic mutations, clinical stage, pathological stage, grade, chemotherapy regimen, surgery type, radiation therapy, receptor phenotype (estrogen receptor [ER], progesterone receptor [PR], HER2) and RCB. Histologic diagnosis of invasive breast carcinoma and HR and HER2 status were obtained on pretreatment core needle biopsy. Cases were considered HR-positive if at least 1% of tumor cells expressed ER and/or PR [30]. HER2 status was determined by immunohistochemistry (IHC) and fluorescent in-situ hybridization (FISH) using American Society of Clinical Oncology (ASCO)/College of American Pathologists (CAP) guidelines [31]. A pCR after NAC was defined as the absence of invasive cancer in the breast and lymph nodes (ypT0/ypTis ypN0).

### Statistical analysis

Demographic and clinical characteristics were summarized with frequencies and percentages. Chi-squared tests were performed to evaluate differences in these characteristics by race. A pCR after NAC was a binary variable (yes/no) defined as the absence of invasive cancer (ypT0/is ypN0) at the time of surgery. Overall survival (OS) was defined as the time from the date of diagnosis to the date of death from any cause. Recurrence-free survival (RFS) was defined as the time from the date of diagnosis to the date of first recurrence. Patients who did not experience a recurrence or death or were lost to follow up at the time of analysis were censored. Within each receptor phenotype, the proportion by race was reported and differences in pCR by race were analyzed with chi-squared tests. Kaplan–Meier curves were used to estimate OS and RFS by race. Log rank tests were used to evaluate differences in OS and RFS by race. Associations between race, pathologic and clinical outcomes were evaluated using multivariable logistic regression and Cox proportional hazard models. All statistical analyses were conducted in SAS version 9.4 (SAS Institute, Inc., Cary, NC, USA) with a significance level of 0.05.

## Results

### Demographic and clinicopathological characteristics

A total of 532 early-stage breast cancer patients treated with NAC followed by surgery were identified (Table 1). The majority of patients were White (60.7%). Black patients comprised 35.3% of the patient population and 3.9% of patients were other races. All tumor receptor phenotypes were included. HR-positive/HER2-negative comprised 36.7%, HER2-positive comprised 36.3%, and triple-negative (TN) comprised 27.1% of the population. The median age was 51.5 years (IQR 44.3, 60.5), ranging from 18.6 to 89.3 years old. The majority of patients were > 40 years of age (86.1%) and had clinical stage II (74.8%) or stage III (19.4%) breast cancer.

**Table 1:** Patient characteristics by race

Characteristic	Black (n = 188)	White (n = 323)	Other (n = 21)	Total (N = 532)	P-value
*Phenotype*					0.55
HER2 +	63 (33.5%)	123 (38.1%)	7 (33.3%)	193 (36.3%)	
HR + /HER2-	66 (35.1%)	121 (37.5%)	8 (38.1%)	195 (36.7%)	
Triple-negative	59 (31.4%)	79 (24.5%)	6 (28.6%)	144 (27.0%)	
*Age*					0.36
> 40 years	162 (86.2%)	280 (86.7%)	16 (76.2%)	458 (86.1%)	
≤ 40 years	26 (13.8%)	43 (13.3%)	5 (23.8%)	74 (13.9%)	
*Clinical stage*					0.96
I	9 (4.8%)	21 (6.5%)	1 (4.8%)	31 (5.8%)	
II	142 (75.5%)	240 (74.3%)	16 (76.2%)	398 (74.8%)	
III	37 (19.7%)	62 (19.2%)	4 (19.0%)	103 (19.4%)	
*Pathological stage*					0.15
0	37 (19.7%)	86 (26.6%)	1 (4.8%)	124 (23.3%)	
I	52 (27.7%)	73 (22.6%)	7 (33.3%)	132 (24.8%)	
II	68 (36.2%)	106 (32.8%)	8 (38.1%)	182 (34.2%)	
III	31 (16.5%)	58 (18.0%)	5 (23.8%)	94 (17.7%)	
*Grade*					0.05
1	8 (4.4%)	29 (9.4%)	2 (10.0%)	39 (7.7%)	
2	89 (49.4%)	154 (50.2%)	5 (25.0%)	248 (48.9%)	
3	83 (46.1%)	124 (40.4%)	13 (65.0%)	220 (43.4%)	
Unknown	8	16	1	25	
*RCB*					0.17
0	36 (19.1%)	88 (27.2%)	2 (9.5%)	126 (23.7%)	
1	28 (14.9%)	39 (12.1%)	5 (23.8%)	72 (13.5%)	
2	78 (41.5%)	130 (40.2%)	10 (47.6%)	218 (41.0%)	
3	46 (24.5%)	66 (20.4%)	4 (19.0%)	116 (21.8%)	
*pCR*					0.03
No	152 (80.9%)	235 (72.8%)	19 (90.5%)	406 (76.3%)	
Yes	36 (19.1%)	88 (27.2%)	2 (9.5%)	126 (23.7%)	
*Anthracycline*					0.48
Yes	123 (65.4%)	195 (60.4%)	14 (66.7%)	332 (62.4%)	
No	65 (34.6%)	128 (39.6%)	7 (33.3%)	200 (37.6)	
*Recurrence*					0.08
Distant	36 (19.1%)	44 (13.6%)	4 (19.0%)	84 (15.8%)	
Local	12 (6.4%)	14 (4.3%)	0 (0.0%)	26 (4.9%)	
Local, Distant	2 (1.1%)	1 (0.3%)	1 (4.8%)	4 (0.8%)	
No Recurrence	138 (73.4%)	264 (81.7%)	16 (76.2%)	418 (78.6%)	

*HR* hormone receptor, *HER2* human epidermal growth factor 2, *pCR* pathologic complete response, *RCB* residual cancer burden

### pCR rates

The pCR rate for all receptor phenotypes in the entire cohort of patients (N = 532) was 23.7%. When comparing these pCR rates among race, a significant difference was found. The pCR in Black patients was 19.1% (*n* = 36), White patients was 27.2% (*n* = 88) and other race/unknown was 9.5% (*n* = 2) (*P* = 0.03). Anthracycline utilization was similar between races (P = 0.48), and when analyzing pCR rates specifically in patients who received anthracycline, no significant difference was seen between races. The pCR rate was evaluated among each receptor phenotype among Black and White patients. Given the small number of patients per each phenotype in the other/unknown category, this group was not included in this analysis (Table 2). No significant difference was seen between Black and White patients with HER2-positive (23.8% vs 35.0%; *P* = 0.12) or HR-positive/HER2-negative breast cancer (7.6% vs 8.3%; *P* = 0.87). However, in patients with TNBC, Black patients had inferior pCR rates compared to White patients (27.1% vs 44.3%; *P* = 0.04). When comparing White versus Black patients in multivariable analysis adjusting for receptor phenotype, White patients demonstrated a higher pCR rate than Black patients with an OR of 1.75 (95% CI 1.10–2.77; *P* = 0.02) (Table 3). No significant difference in pCR was noted when comparing patients of other/unknown race versus Black patients (OR 0.45 [95% CI 0.10–2.06]).

**Table 2:** The pCR rate by phenotype and race

Phenotype	No pCR (n = 387)	pCR (n = 124)	P-value
*Triple-Negative*			0.04
Black	43 (72.9%)	16 (27.1%)	
White	44 (55.7%)	35 (44.3%)	
*HER2* +			0.12
Black	48 (76.2%)	15 (23.8%)	
White	80 (65.0%)	43 (35.0%)	
*HR* + */HER2-*			0.87
Black	61 (92.4%)	5 (7.6%)	
White	111 (91.7%)	10 (8.3%)	

*HR* hormone receptor, *HER2* human epidermal growth factor 2, *pCR* pathologic complete response

**Table 3:** Multivariable logistic regression model of race on pCR adjusted for phenotype

Effect	Odds ratio	95% CI	P-value
*Race*			0.02
White vs Black	1.75	1.10–2.77	
Other vs Black	0.45	0.10–2.06	
*Phenotype*			< .001
HER2 + vs triple-negative	0.72	0.45–1.14	
HR + /HER2- vs triple-negative	0.15	0.08–0.28	

*HR* hormone receptor, *HER2* human epidermal growth factor 2, *pCR* pathologic complete response

### Survival outcomes

**Fig. 1:**
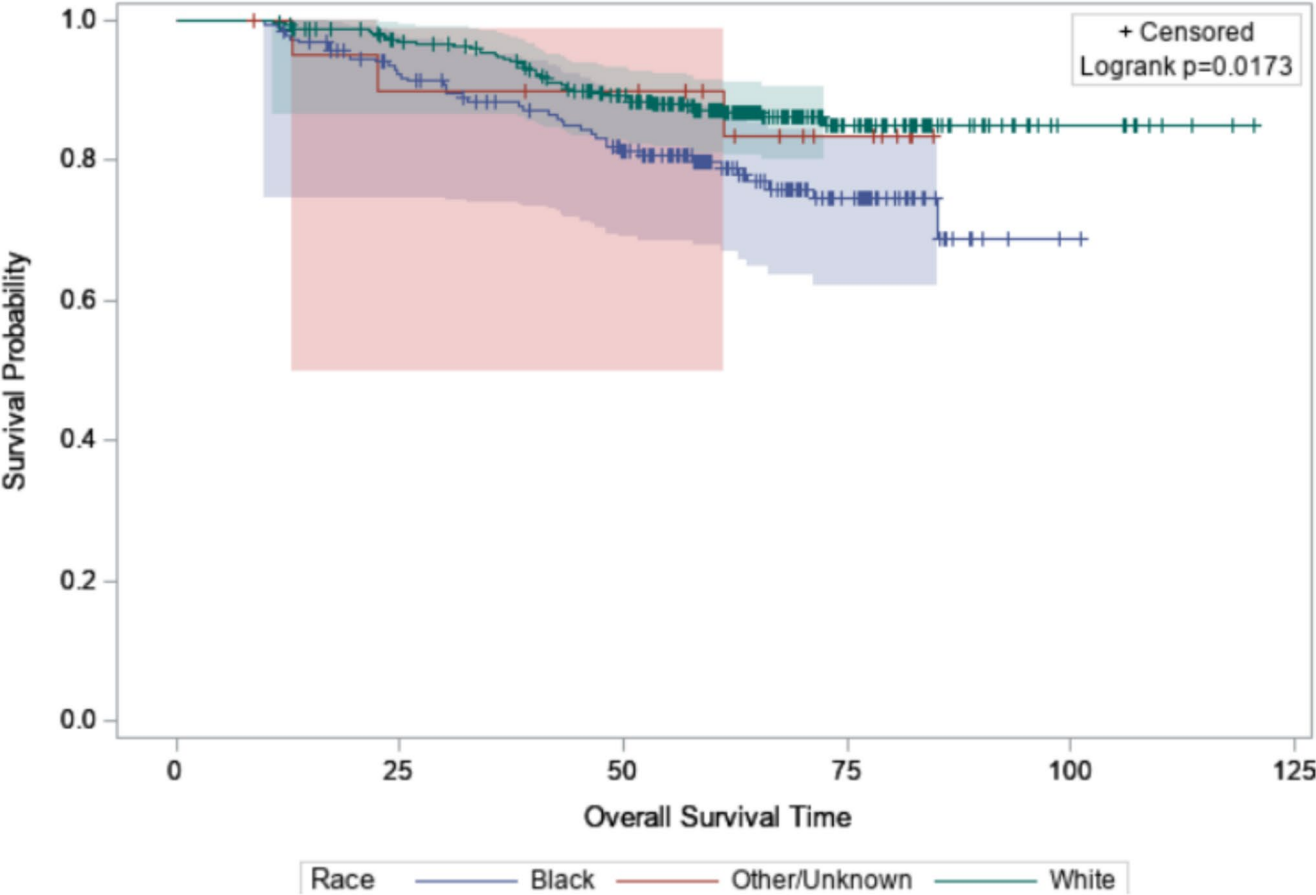
Overall survival by race (all receptor phenotypes)

**Fig. 2:**
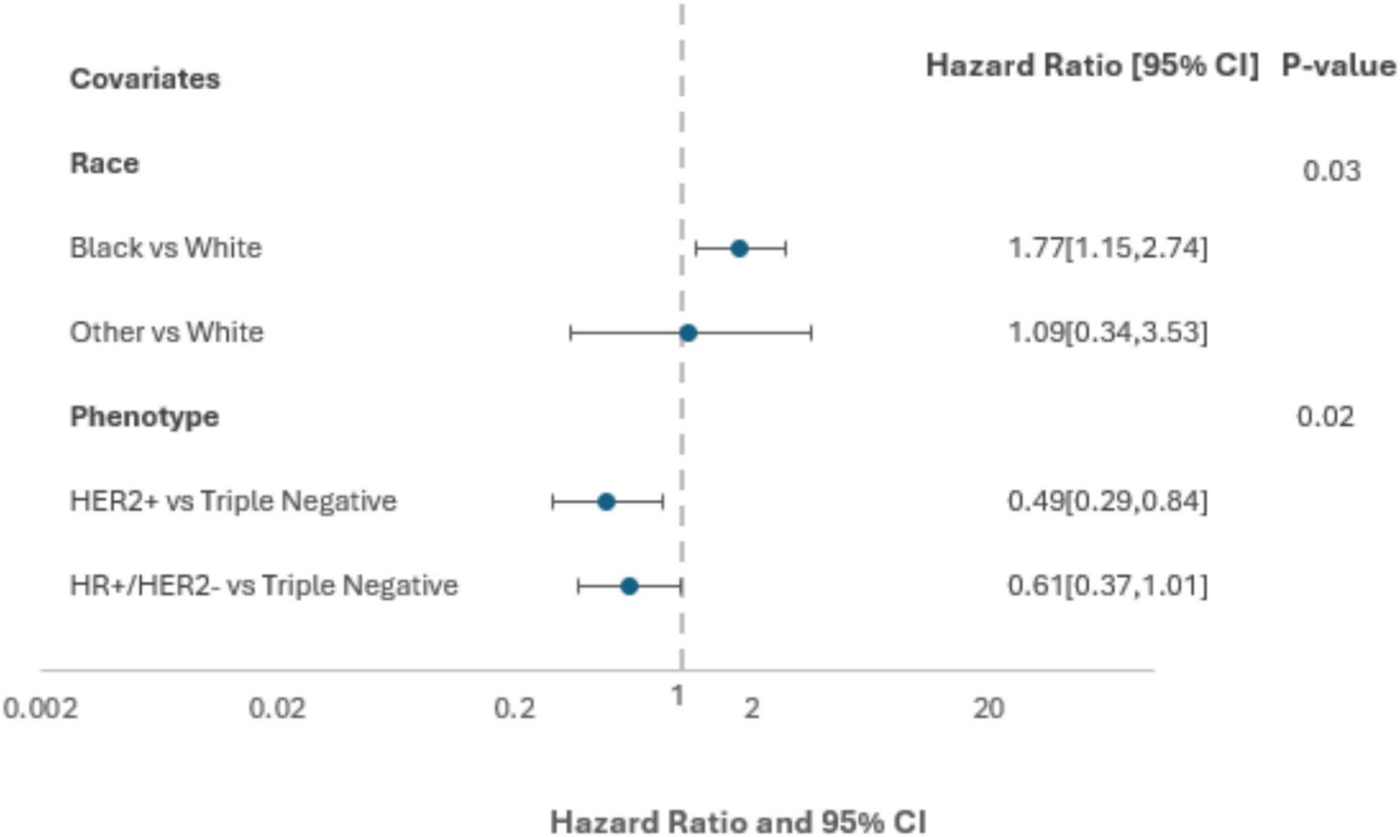
Multivariable cox proportional hazards model of race on overall survival adjusted for phenotype. *HR* hormone receptor, *HER2* human epidermal growth factor 2

The median follow-up for all patients was 65 months. OS was significantly different among the races when evaluating the entire cohort (*P* = 0.02) (Fig. 1). At 60 months, the OS rate in Black patients was 79.9% (95% CI 67.8–87.9) and for White patients it was 87.3% (95% CI 81.1–91.4). When adjusting for receptor phenotype using a multivariable Cox proportions hazard model, OS remained inferior in Black patients when compared to White patients (HR 1.77 [95% CI 1.15–2.74]; *P* = 0.03) (Fig. 2). No significant difference was seen when comparing patients of other/unknown race to White patients (HR 1.09 [95% CI 0.34–3.53]).

**Fig. 3:**
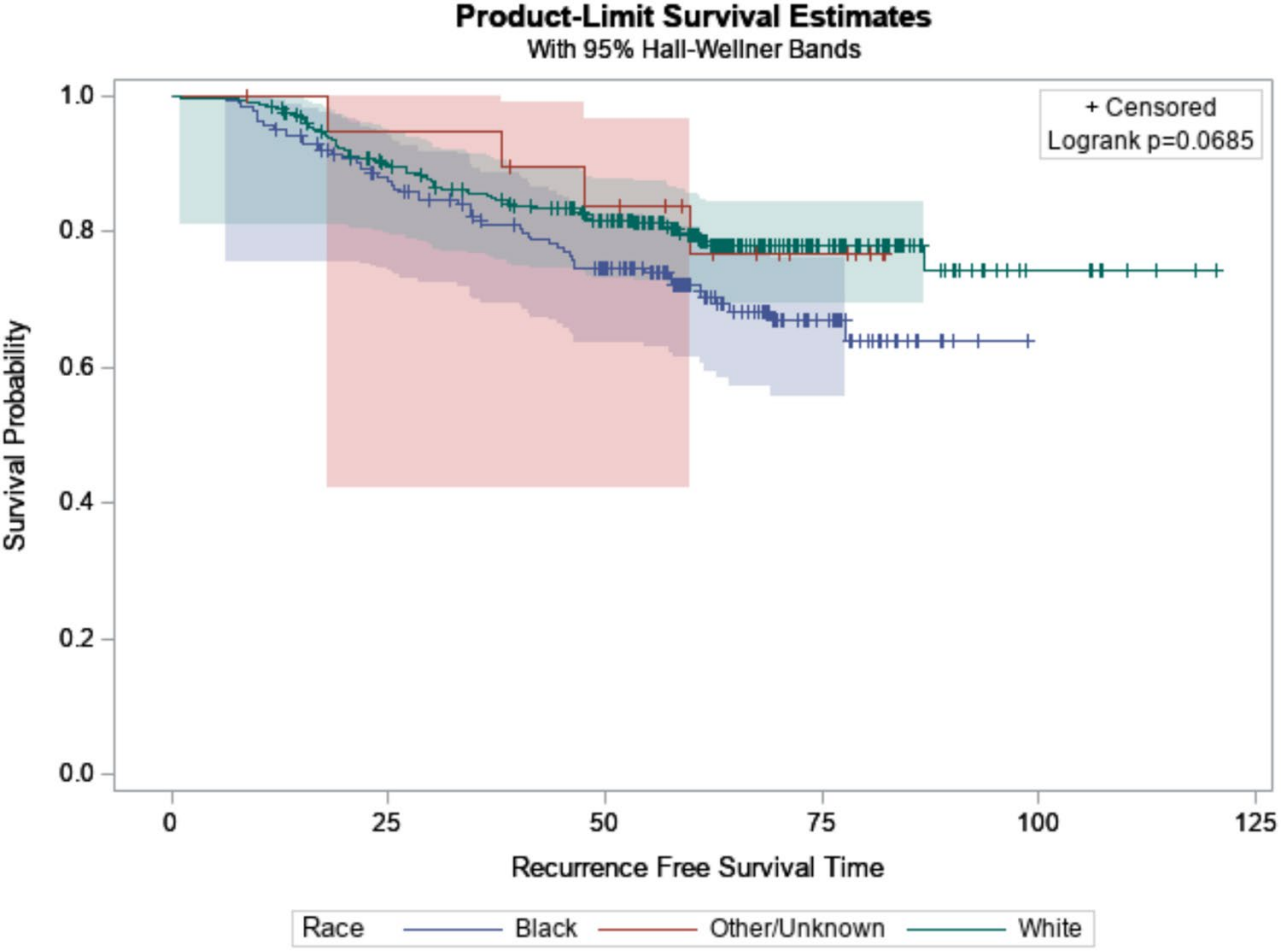
Recurrence free survival by race (all receptor phenotypes)

The RFS was not significantly different among the different races (Fig. 3). This remained true when evaluating all the receptor phenotypes together (*P* = 0.07), and when analyzing each individually. At 60 months, the RFS probability for Black patients was 72.3% (95% CI 61.4–80.6) and 79.4% (95% CI 71.0–85.7) for White patients.

## Discussion

Identifying factors that impact pCR is crucial because the response to NAC and amount of RCB at the time of surgery are well-established predictors of outcomes, as we have shown from our previous publications with the same dataset [13, 18]. It is important to note that the pCR rates have increased in recent years, especially in patients with TNBC. The patients identified in this study were all treated prior to 2017 and are similar to other published pCR data in this era [5, 6, 10].

In this single-institution cohort of women receiving NAC for early-stage breast cancer, pCR and OS rates were inferior among Black patients compared to White patients when analyzing the entire population (including HR-positive, HER2-positive, and TNBC). Other studies have reported similar findings, including a study of 690 early-breast cancer patients by Zhao et al., which demonstrated worse pCR rates in Black patients compared to White patients (28.6% versus 36.6%; *P* = 0.4) [10]. Those patients not obtaining a pCR had worse OS (aHR 6.10 [95% CI 2.80–13.32].

When evaluating pCR rates within each receptor subtype by race, we found no difference in the pCR rates in the HER2-positive subgroup or HR-positive/HER2-negative subgroup. This is in contrast to other published literature [10, 27, 28]. For example, Kyalwazi et al., analyzed 974 patients with high-risk breast cancer who had received neoadjuvant chemotherapy. Although results demonstrated inferior distant RFS in Black patients (compared to White patients) if a pCR was not obtained, when they analyzed the percentage of patients who achieved a pCR, there was no difference in pCR rates between races, regardless of subtype [28]. Further highlighting the heterogeneity of published data according to pCR and its association with race, a study by Zhao et al., reported results differing from both Kyalwazi et al. and our results. Zhao et al. demonstrated inferior pCR rates among Black patients with an HR-negative/HER2-positive subtype when compared to White patients (aOR 0.30 [95% CI 0.11–0.81]) [10]. To further their investigation, the HR-negative/HER2-positive tumors were evaluated on a molecular level. This showed Black patients were more likely to have an alteration in the MAPK pathway, which can confer resistance to HER2-targeted therapies, making them less effective [32]. Although our study did not focus on molecular characteristics, it is important to note that racial genetic and epigenetic differences may play a role in treatment response and outcomes and deserve continued investigation.

Our study demonstrated lower pCR rates in Black patients compared to White patients with TNBC, which is similar to a large retrospective analysis of the National Cancer Database (NCDB) from 2010 to 2019 [5]. In this study, pCR rates and outcomes were evaluated in 40,890 women with early-stage TNBC. Black patients with TNBC were less likely to achieve a pCR compared to White patients (OR 0.89 [95% CI 0.83–0.95], *P* = 0.001). If they did achieve a pCR, however, the 5-year OS rates were quite similar between Black and White patients (0.91 [95% CI 0.89–0.92]) versus (0.92 [95% CI 0.909–0.922]), respectively. In another NCDB analysis of 27,300 patients, inferior pCR rates in Black patients with TNBC was again demonstrated (37% versus 43%, *P* < 0.001) [27]. These studies, including our study, were all performed prior to the incorporation of immunotherapy into the neoadjuvant regimen for patients with TNBC. With the addition of pembrolizumab to chemotherapy, based on the KEYNOTE-522 clinical trial results, pCR rates in TNBC patients have significantly increased and currently approximate 65% [21]. In a NCDB analysis evaluating use of immunotherapy in patients with TNBC from 2017 to 2021, incorporation of immunotherapy in the neoadjuvant setting increased from 4.2% in 2017 to 48.0% in 2021 [29]. Additionally, this study showed that among those receiving immunotherapy, there was no difference in pCR rate by race. It will be interesting to continue investigating pCR rates in patients receiving chemoimmunotherapy to assess if racial differences still exist among patients with TNBC or if the increased use of immunotherapy narrows this gap.

The RFS was not significantly different among the races (*P* = 0.07), although numerically the RFS at 5 years was inferior for Black patients compared to White patients (72.3% versus 79.4%). This is important to recognize, as it indicates there are other variables playing a role in survival. One variable we must take into consideration would be late complications from anthracyclines. However, anthracycline use was similar between races in our study, and therefore, anthracycline-induced heart failure or leukemias are not likely responsible for the inferior OS without inferior RFS.

Our study focused specifically on race and its association with outcomes. However, it is important to recognize other factors that may influence pCR and outcomes, such as age, clinical trial enrollment, and time from diagnosis to treatment. In a study utilizing the Chicago Multiethnic Epidemiologic Cohort of Breast Cancer (ChiMEC) database, 2196 women treated with NAC were identified [6]. After adjusting for subtype, grade, and stage, young (defined as ≤ 40 years) Black women were less likely to achieve a pCR compared to young White women (aOR 0.41 [95% CI 0.19–0.88] *P* < 0.001). Young Black women were also less likely to enroll in clinical trials compared to young White women (8.5% versus 19.6%, respectively). The pCR rate was not statistically different when comparing those who enrolled on a clinical trial versus those who did not, but there was improvement in DFS (*P* = 0.022) and OS (*P* = 0.036) in patients enrolled on clinical trials. The time from diagnosis to treatment initiation could be another contributing factor to rates of pCR. In a study by Shubeck et al., patients who initiated NAC within 31 days of diagnosis were more likely to achieve a pCR, suggesting the potential impact of initiating NAC in a timely manner [33]. Perhaps this could play a role in decreased pCR rates seen in young Black women reported in the analysis if the CHiMEC database. In that analysis, young Black women were more likely to have a longer time before initiation of NAC compared to young White women (41 vs 33 days; *P* = 0.004) [6]. This discrepancy was also shown by Killelea et al., with similar time to treatment (Blacks 41.3 days versus Whites 32.7 days, respectively; *P* < 0.001) [27]. No specific factor has been identified as the cause for longer time to therapy among Black women. Longer time to therapy is most likely multifactorial and due to socioeconomic differences as well as inherent barriers within healthcare systems. Further defining these differences and modifiable barriers within the healthcare system is vital to narrow this gap between races.

This study has several limitations including its retrospective nature and small sample size. This was a single institution review, which limits its generalizability. Our study focused specifically on the impact of race on outcomes, but more factors exist that can impact outcomes including age, clinical trial enrollment, incorporation of immunotherapy, time to NAC and other socioeconomic factors such as income level and education. Lastly, we did not investigate molecular features, which should be incorporated into further investigations given biologic differences can clearly play a role in outcomes.

## Conclusion

In conclusion, our study demonstrated that racial disparities exist among patients with early-stage breast cancer receiving NAC. This difference is apparent in pCR rates and OS. An awareness exists of socioeconomic barriers that could play a role in these disparities, as well as differences in biology. Future investigation of these potential factors is needed. We must take critical steps to address the modifiable factors within the healthcare system to improve racial disparities in breast cancer outcomes.

## Data Availability

The datasets generated during the current study are available from the corresponding author on reasonable request.
